# Efficacy and safety of scalp acupuncture in improving neurological dysfunction after ischemic stroke: A protocol for systematic review and meta-analysis

**DOI:** 10.1097/MD.0000000000021783

**Published:** 2020-08-21

**Authors:** Lei Sun, Yihua Fan, Wei Fan, Jing Sun, Xia Ai, Haifa Qiao

**Affiliations:** a Shaanxi University of Chinese Medicine, Shaanxi Province; b Tianjin University of Traditional Chinese Medicine, Tianjin, China.

**Keywords:** ischemic stroke, meta-analysis, neurological dysfunction, scalp acupuncture, systematic review

## Abstract

**Background:**

Scalp acupuncture is remarkable in improving neurological dysfunction of ischemic stroke patients. This study aims to systematically evaluate the efficacy and safety of scalp acupuncture in improving neurological dysfunction of ischemic stroke patients.

**Methods:**

Randomized controlled trials of scalp acupuncture against ischemic stroke patients will be searched in PubMed, Web of Science, Embase, Cochrane Library, the Chongqing VIP Chinese Science and Technology Periodical Database, Chinese Biological and Medical database, China National Knowledge Infrastructure, and Wanfang database from inception to July, 2020. Two researchers will perform data extraction and risk of bias assessment independently. Statistical analysis will be conducted in RevMan 5.3.

**Results:**

This study will summarize the present evidence by exploring the efficacy and safety of scalp acupuncture in improving neurological dysfunction in ischemic stroke patients.

**Conclusions:**

The findings of the study will help to determine potential benefits of scalp acupuncture against ischemic stroke at different stage.

**OSF Registration number:**

DOI 10.17605/OSF.IO/T26P8.

## 1 Introduction

Ischemic stroke, also known as cerebral infarction, caused by blockage or narrowing in the arteries supplying blood and oxygen to the brain, is a common and frequently-occurring diseases that threaten human health, with high mortality, high disability, and high recurrence.^[1–3]^ It is characterized by focal neurological dysfunction such as hemiplegia and aphasia. At present, the main treatment methods of ischemic stroke include thrombolytic therapy, intravascular interventional therapy, antiplatelet, anticoagulation, defibrillation, and neuroprotection. Although timely thrombolytic therapy could reduce nerve cell death and injury to a certain extent, the limited time window and strict requirements for thrombolysis have led to the lower clinical utilization of thrombolytic therapy.^[4,5]^ Moreover, there are still 2/3 patients have different degrees of disability after successful intravenous thrombolysis,^[6]^ which makes it important to improve neurological dysfunction recovery after ischemic stroke.

Acupuncture therapy has been applied to treat ischemic stroke for thousands years in China, and nowadays it has been widely used in many other countries all over the world. Clinical studies have shown that acupuncture therapy could not only effectively promote neurological function recovery after ischemic stroke,^[7,8]^ but also improve patients with post-stroke depression, aphasia, and dysphagia.^[9,10]^ It has been proved that acupuncture could reduce the disability rate, and improve living quality of patients. The mechanism might be involved with anti-oxidative stress, anti-inflammation, scavenging free radicals, inhibiting apoptosis, and promoting the establishment of cerebral collateral circulation after ischemia.^[11–13]^

Scalp acupuncture, also known as head acupuncture, is a combined treatment based on traditional acupuncture and neurology. It could stimulate the brain neurons of the underlying area related to the impaired functions by inserting needles into loose areolar tissue layer of the scalp. Scalp acupuncture is a commonly used therapy against neurological disorders, such as paralysis, Parkinson disease, and multiple sclerosis.^[14–16]^ It could encourage recruitment of healthy brain cells to perform the lost function. Nowadays, scalp acupuncture is remarkable in improving neurological dysfunction in ischemic stroke patients.^[17,18]^ However, whether the efficacy and safety of scalp acupuncture in improving neurological function against ischemic stroke is superior to other therapies is currently lacking evidence of systematic review and meta-analysis. This study aims to systematically evaluate the efficacy and safety of scalp acupuncture in improving neurological dysfunction of ischemic stroke patients.

## 2 Methods

### 2.1 Study registration

This protocol of systematic review and meta-analysis has been drafted under the guidance of the preferred reporting items for systematic reviews and meta-analyses protocols. Moreover, it has been registered on open science framework on July 15, 2020. (Registration number: DOI 10.17605/OSF.IO/T26P8).

### 2.2 Ethics

Ethical approval is not required because there is no patient recruitment and personal information collection, and the data included in our study are derived from published literature.

### 2.3 Inclusion criteria for study selection

#### 2.3.1 Type of studies

Randomized controlled trials (RCTs) of scalp acupuncture in improving neurological dysfunction of ischemic stroke patients will be included. The language will be limited to Chinese and English.

#### 2.3.2 Type of participants

All the included cases conform to the diagnosis of ischemic stroke,^[19]^ regardless of nationality, race, age, gender, and source of cases.

#### 2.3.3 Type of interventions

The study focuses on RCTs of ischemic stroke with scalp acupuncture therapy versus other acupuncture, sham acupuncture or western medicine.

#### 2.3.4 Type of outcome measures

The outcome measures are Fugl-Meyer assessment (including Brunnstrom-Fugl-Meyer), Barthel index score (including the modified Barthel index), Neurological Deficit Scor (including NHI stroke scale and Chinese version of NIH stroke scale), and occurrence of adverse events.

### 2.4 Exclusion criteria

(1) Studies with the treatment group using scalp acupuncture combined with other treatments will be excluded;
(2) Studies with unsatisfactory outcome indicators;
(3) As for duplicate published literatures, select the literature with the most complete data;
(4) Missing main content, unable to conduct statistical analysis, and unable to obtain the literature after contacting the author;
(5) Literature with errors in random methods.

### 2.5 Search strategy

The protocol of this systematic review and meta-analysis should be complied with the preferred reporting items for systematic review and meta-analysis protocols statement guidelines.^[20]^ The following 8 databases will be retrieved for all relevant RCTs: PubMed, Web of Science, Embase, Cochrane Library, the Chongqing VIP Chinese Science and Technology Periodical Database, Chinese Biological and Medical database, China National Knowledge Infrastructure, and Wanfang database. The reference lists of relevant articles will be reviewed for eligible inclusion.

The subject terms and combination of keywords as, scalp acupuncture and ischemic stroke will be used in the search. The search terms will be revised according to the rule of each database. The search strategy of PubMed is listed in Table 1.

**Table 1:**
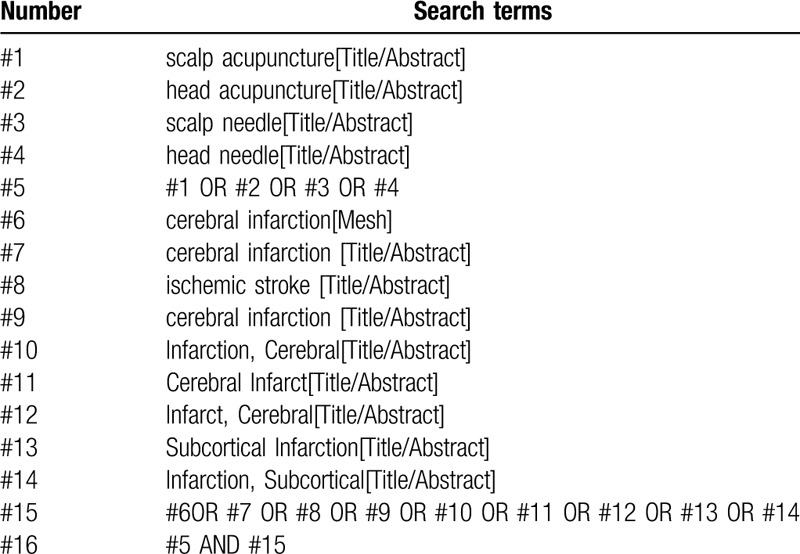
Search strategy in PubMed database.

### 2.6 Data extraction

**Figure 1:**
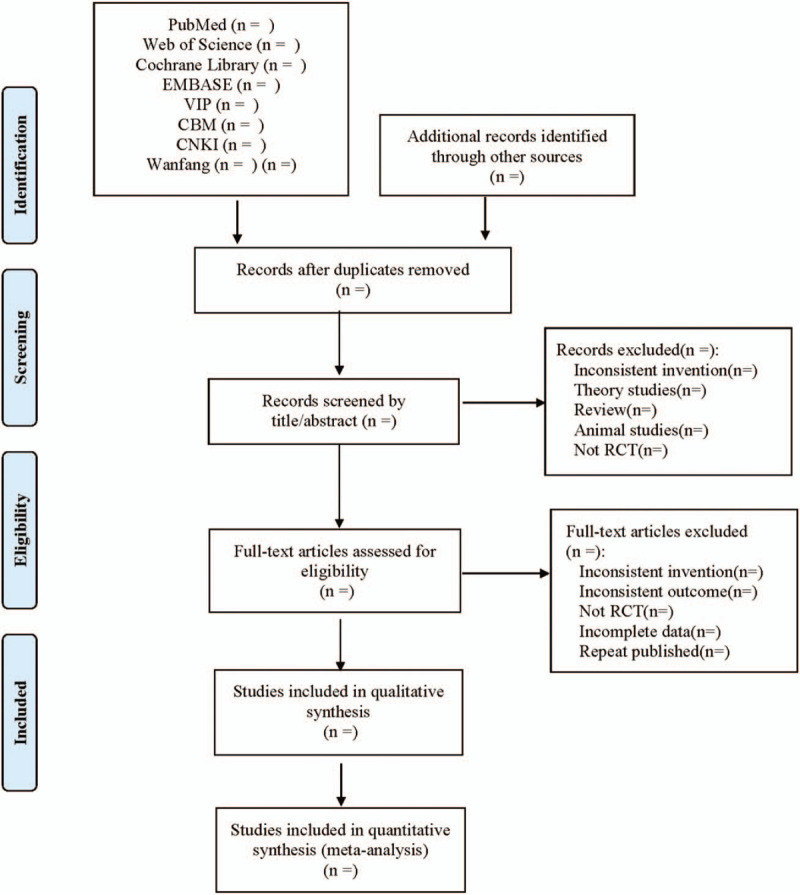
Flow diagram.

Endnote X7 will be used for literature management. All relevant articles of full text that meet these criteria will be obtained. In case of different opinions, a third reviewer will make the final decision. The process of study selection is shown in a flow diagram (Fig. 1). Two reviewers will separately complete the extraction. Under the instruction of the Cochrane Collaboration, Excel 2019 is used to set up a data extraction table to extract data, including study identification (title, authors, journal, publication year, and country), participants information (age, sample size, sex ratio, course of disease), randomization method, concealment, interventions in treatment and control groups, outcomes, adverse events, and other details. Divergence will be solved by consulting and discussing with the third author.

### 2.7 Risk of bias assessment

Two researchers independently will evaluate the risk of bias in RCTs in accordance with the Cochrane Handbook of Systematic Reviewers, including the following items: random sequence generation, allocation concealment, blinding of participants and personnel, blinding of outcome assessment, incomplete outcome data, selective reporting, and other bias. The quality of studies is classified as being at of high, unclear or low risk of bias. In case of disagreement, a third researcher will decide.

### 2.8 Statistical analysis

#### 2.8.1 Data synthesis

The RevMan 5.3 software provided by the Cochrane Collaboration will be used for statistical analysis. ① Relative risk (RR) is selected as the statistic for the dichotomous variable. For continuous variables, weighted mean difference is selected when the tools and units of measurement indicators are the same, standardized mean difference is selected with different tools or units of measurement, and all the above are represented by effect value and 95% confidence interval. ② Heterogeneity test: *Q* test is used to qualitatively determine inter-study heterogeneity. If *P* ≥ .1, there is no inter-study heterogeneity; If *P* < .1, it indicate inter-study heterogeneity. At the same time, *I*^*2*^ value is used to quantitatively evaluate the inter-study heterogeneity. If *I*^*2*^ ≤ 50%, the heterogeneity is considered to be good, and the fixed-effect model is adopted. If *I*^*2*^ > 50%, it is considered to be significant heterogeneity, the source of heterogeneity will be explored through subgroup analysis or sensitivity analysis. If there is no obvious clinical or methodological heterogeneity, it will be considered as statistical heterogeneity, and the random-effect model will be used for analysis. Descriptive analysis will be used if there is significant clinical heterogeneity between the 2 groups and subgroup analysis is not available.

#### 2.8.2 Dealing with missing data

If data is missing or incomplete, we will contact the corresponding author to obtain the missing data. If not, this study will be removed.

#### 2.8.3 Heterogeneity and subgroup analysis

In order to reduce the clinical heterogeneity between studies, subgroup analysis is conducted according to the course of disease, which will be divided into acute state, recovery state, and sequelae stage.

#### 2.8.4 Sensitivity analysis

In order to test the stability of meta-analysis results of indicators, a one-by-one elimination method will be adopted for sensitivity analysis. Subgroup analysis is conducted according to the course of treatment and the course of disease.

#### 2.8.5 Reporting bias

For the major outcome indicators, if the included study is ≥10, funnel plot will be used to qualitatively detect publication bias. Egger and Begg test are used to quantitatively assess potential publication bias.

#### 2.8.6 Evidence quality evaluation

The grading of recommendations assessment, development, and evaluation^[23]^ will be used to assess the quality of evidence. It contains 5 domains (bias risk, consistency, directness, precision, and publication bias). And the quality of evidence will be rated as high, moderate, low, and very low.

## 3 Discussion

Scalp acupuncture is a special acupuncture method developed in recent decades. It is a modern innovation and development of traditional Chinese meridian theory and the reflex somatotopic system organized on the surface of the scalp in Western medicine. By inserting acupuncture needles subcutaneously into specific area on the scalp corresponding to the cortical areas of the cerebrum responsible for central nervous system functions such as motor function, sensory input, speech, hearing, and balance,^[21]^ it could function in therapeutic effect for treating acute and chronic central nervous system disorders.^[22–25]^ Clinical research shows that scalp acupuncture on motor area could expand cerebral blood vessels, increase cerebral blood flow, reduce infarct focus, and promote the establishment of cerebral collateral circulation to improve motor function in ischemic stroke.^[17,26]^ The mechanism might be that after stimulating the reflex somatotopic system, the bioelectric effect is transmitted to the cerebral cortex through meridians and nerves to change the excitability of cerebral cortical nerve cells and accelerate the establishment of cerebral collateral circulation.

Yet, the efficacy and safety of scalp acupuncture in improving neurological function against ischemic stroke still needs evidence of systematic review and meta-analysis. We try to conduct this systematic review tend to figure out the efficacy and safety of scalp acupuncture for ischemic stroke. This meta-analysis and systematic review will help to determine potential benefits of scalp acupuncture against ischemic stroke at different stage. However, the study has some limitations. Our search did not include studied in other languages except Chinese and English, which might result in certain selective bias. In addition, due to the different acupoints used in the treatment of ischemic stroke by scalp acupuncture, there might be some heterogeneity.

## Author contributions

**Data collection:** Lei Sun and Yihua Fan

**Funding support:** Xia Ai and Haifa Qiao

**Literature retrieval:** Wei Fan.

**Software operating:** Lei Sun and Jing Sun.

**Supervision:** Xia Ai and Haifa Qiao

**Writing – original draft:** Lei Sun and Yihua Fan.

**Writing – review & editing:** Xia Ai and Haifa Qiao.
